# MI-PACE Home-Based Cardiac Telerehabilitation Program for Heart Attack Survivors: Usability Study

**DOI:** 10.2196/18130

**Published:** 2021-07-08

**Authors:** Eric Y Ding, Nathaniel Erskine, Wim Stut, David D McManus, Amy Peterson, Ziyue Wang, Jorge Escobar Valle, Daniella Albuquerque, Alvaro Alonso, Naomi F Botkin, Quinn R Pack, David D McManus

**Affiliations:** 1 Division of Cardiology Department of Medicine University of Massachusetts Medical School Worcester, MA United States; 2 Department of Anesthesiology Duke University Durham, NC United States; 3 Philips Research Eindhoven Netherlands; 4 University of Massachusetts Memorial Marlborough Hospital Marlborough, MA United States; 5 MaineHealth Cardiology Maine Medical Center Augusta, ME United States

**Keywords:** cardiac rehabilitation, telerehabilitation, health watch, mHealth, exercise

## Abstract

**Background:**

Cardiac rehabilitation programs, consisting of exercise training and disease management interventions, reduce morbidity and mortality after acute myocardial infarction.

**Objective:**

In this pilot study, we aimed to developed and assess the feasibility of delivering a health watch–informed 12-week cardiac telerehabilitation program to acute myocardial infarction survivors who declined to participate in center-based cardiac rehabilitation.

**Methods:**

We enrolled patients hospitalized after acute myocardial infarction at an academic medical center who were eligible for but declined to participate in center-based cardiac rehabilitation. Each participant underwent a baseline exercise stress test. Participants received a health watch, which monitored heart rate and physical activity, and a tablet computer with an app that displayed progress toward accomplishing weekly walking and exercise goals. Results were transmitted to a cardiac rehabilitation nurse via a secure connection. For 12 weeks, participants exercised at home and also participated in weekly phone counseling sessions with the nurse, who provided personalized cardiac rehabilitation solutions and standard cardiac rehabilitation education. We assessed usability of the system, adherence to weekly exercise and walking goals, counseling session attendance, and disease-specific quality of life.

**Results:**

Of 18 participants (age: mean 59 years, SD 7) who completed the 12-week telerehabilitation program, 6 (33%) were women, and 6 (33%) had ST-elevation myocardial infarction. Participants wore the health watch for a median of 12.7 hours (IQR 11.1, 13.8) per day and completed a median of 86% of exercise goals. Participants, on average, walked 121 minutes per week (SD 175) and spent 189 minutes per week (SD 210) in their target exercise heart rate zone. Overall, participants found the system to be highly usable (System Usability Scale score: median 83, IQR 65, 100).

**Conclusions:**

This pilot study established the feasibility of delivering cardiac telerehabilitation at home to acute myocardial infarction survivors via a health watch–based program and telephone counseling sessions. Usability and adherence to health watch use, exercise recommendations, and counseling sessions were high. Further studies are warranted to compare patient outcomes and health care resource utilization between center-based rehabilitation and telerehabilitation.

## Introduction

Cardiac rehabilitation provides longitudinal cardiopulmonary exercise training with additional disease management interventions to patients with cardiovascular diseases [1,2]. Participation in cardiac rehabilitation is guideline-recommended after many acute cardiovascular events because it can lower cardiovascular mortality, reduce hospital readmissions, and improve quality of life [3,4]. However, many eligible patients never receive referrals for cardiac rehabilitation, and a high proportion of patients who receive referrals never enroll [5-10]. This may be due to numerous factors, such as cost of enrollment, lack of motivation, or inadequate patient education regarding the benefits of cardiac rehabilitation [2]. In a scientific statement from the American Association of Cardiovascular and Pulmonary Rehabilitation, the American Heart Association, and the American College of Cardiology in 2019 [2], it was suggested that less than 1 in 6 patients participated in cardiac rehabilitation after hospitalization for myocardial infarction. Diverse patient, community, health care provider, hospital system, and insurance factors contribute to the persistent underutilization of cardiac rehabilitation [5-7,11]. Typically, outpatients perform cardiac rehabilitation in a dedicated facility under clinician supervision with limited scheduling hours. Difficulties in scheduling, traveling, and financing center-based cardiac rehabilitation help to drive poor enrollment and retention in cardiac rehabilitation [6,12].

Delivery of cardiac rehabilitation using contemporary telecommunication and smart device technologies (cardiac telerehabilitation, ie, tele-CR) may reduce logistical and financial barriers associated with cardiac rehabilitation by facilitating cardiac rehabilitation in the home [13-16]. The ability to perform cardiac rehabilitation at home may increase participation while providing comparable outcomes for patient health-related quality of life, exercise capacity, and mortality [17-21]. Consumers increasingly use internet-connected mobile and wearable devices to monitor fitness [22]; therefore, they may find tele-CR preferable to center-based cardiac rehabilitation. Furthermore, telehealth platforms can also potentially compound and extend the clinical effectiveness of cardiac rehabilitation for patients who have completed ambulatory center-based programs [23].

Despite the widespread need for rehabilitation after acute myocardial infarction and technological innovations in this space, wearable device–based rehabilitation solutions are still being studied for evidence to support their adoption and use, though recent developments have significantly advanced this area of research [23-26]. The development of a user-centered platform that is acceptable to patients and can impact key clinical or patient-reported outcomes will likely be a critical component of the clinical adoption of tele-CR [14]. In this study, we examined the usability of and adherence to a nurse-supported 12-week telerehabilitation intervention after acute myocardial infarction for patients who declined to participate in conventional rehabilitation. We conducted exploratory analyses to also examine changes in disease-specific quality of life rehabilitation.

## Methods

### Study Setting and Sample

**Figure 1:**
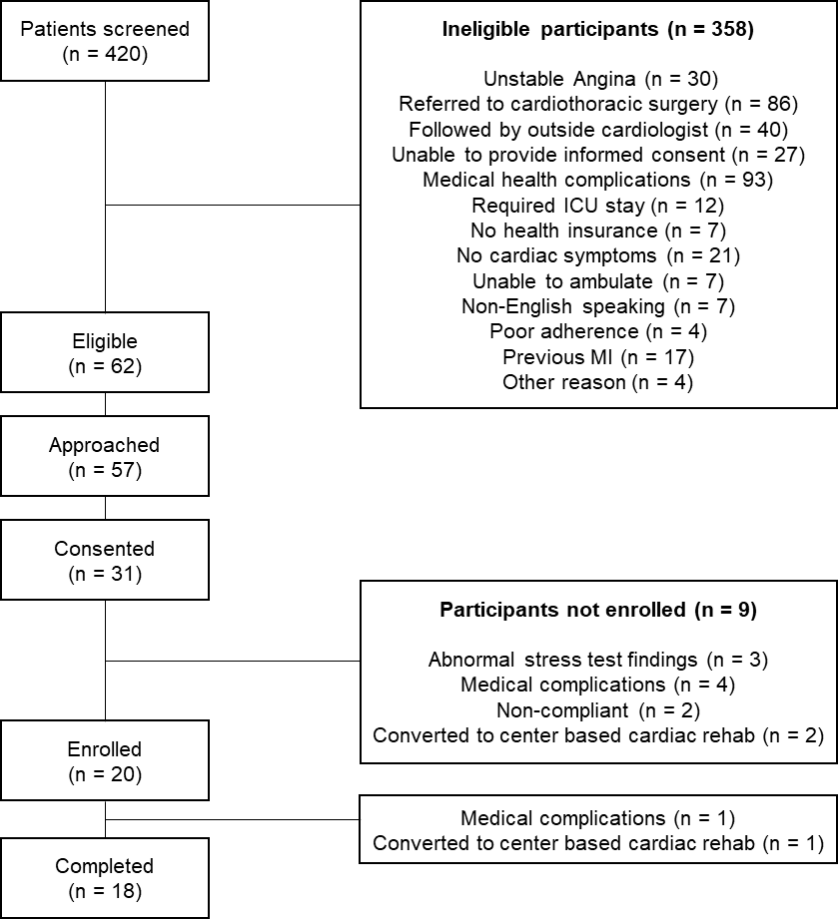
Recruitment flow diagram. MI: myocardial infarction; ICU: intensive care unit.

All adults who were hospitalized at a single tertiary academic medical center in central Massachusetts for acute myocardial infarction between June and November 2018 were screened for eligibility by trained research assistants using International Classification of Disease Tenth Revision codes, problem lists, laboratory results, and electrocardiogram (ECG) findings, as validated in other studies [27]. Patients between 40 and 80 years, fluent and literate in English, meeting clinical indications for cardiac rehabilitation, and with access to an environment or facilities to perform exercise were eligible for inclusion (Figure 1). Patients who preferred to enroll in conventional center-based cardiac rehabilitation, planned to receive follow-up cardiovascular care outside of our hospital system, were unable to participate in follow-up sessions, had subsequent myocardial infarction, had no health insurance, were unable to provide informed consent, were unable to ambulate, were unable to adhere to study protocols, had unstable angina, had serious medical conditions that precluded study participation (for example, cancer), had no symptoms at the time of their presentation for myocardial infarction, underwent cardiac surgery, and were staying in the intensive care unit were excluded. We only targeted patients who chose to forego traditional, center-based cardiac rehabilitation to provide an alternative cardiac rehabilitation delivery modality; if a patient was deemed appropriate for center-based cardiac rehabilitation by their primary medical team, and agreed to enroll, we opted to avoid interfering with their prescribed management. Research assistants approached potential participants who had acute myocardial infarctions (and their care teams) prior to their discharge. Participants provided written informed consent for study participation and received training on study devices and procedures.

### Study Overview

Each patient obtained a stress test within 4 weeks of screening and enrollment. At an in-person baseline visit, each participant received a watch and tablet, as well as device training. The baseline assessment during their index hospitalization included questions on perceptions of cardiac rehabilitation and disease-specific quality of life. Per standard of care for conventional cardiac rehabilitation, each participant needed to complete a clinically indicated stress test within 4 weeks of hospital discharge to assess safety for exercise and establish target heart rates for rehabilitation activities. A Bruce protocol [28] was followed, and baseline ECG, heart rate, and blood pressure were closely monitored prior to, during, and after exercise. This exercise test is divided into successive stages of increasing intensity, and patients are asked about symptoms throughout. A report is generated upon conclusion of the test and interpreted by medical staff—physician (author DDM) screened all exercise test results for abnormal findings that might place participants at risk from participation in cardiac rehabilitation; participants with concerning results were excluded from the study and referred to follow up with their treating physician. Participants with no abnormal stress test findings attended the in-person training session, during which a trained study staff member provided oral and written instructions for telerehabilitation activities at home.

### Components of the Telerehabilitation Program

The MI-PACE tele-CR program included a validated wearable device (Health Watch [29]; Philips Healthcare) that was connected via Bluetooth to an Android tablet computer (Tab 4; Lenovo Group Limited). An app displayed goals and progress for exercise and walking. There was a dashboard for the cardiac rehabilitation nurse to enter the goals and to view the progress of the patients. Counseling and education sessions with the nurse were scheduled weekly over the 12-week study period.

### Cardiac Rehabilitation Methods

Research staff instructed participants to complete a set number of walking sessions (light intensity) consisting of bouts of at least 2 minutes, and moderate to vigorous intensity exercise sessions. Rehabilitation counseling sessions were designed by the cardiac rehabilitation nurse and cardiologist (authors AP and DDM, respectively) a priori and were standardized to include components from center-based cardiac rehabilitation programs. Each participant’s target heart rate range was set by the cardiac rehabilitation nurse based on the participant’s resting and maximum heart rate and performance on the exercise stress test in accordance with conventional cardiac rehabilitation practices [30]. The number and duration of prescribed exercise and walking sessions was determined based on (1) the participant’s level of physical activity prior to their acute myocardial infarction, (2) the participant’s level of activity after the acute myocardial infarction, (3) exertional chest pain after their cardiac event, (4) orthopedic limitations, and (5) the participant’s perceptions of their difficulty achieving exercise target goals. Weekly goals were subject to modification based on the participant’s performance the preceding week. Successful completion of goals prompted an increase in the frequency or duration of the sessions, whereas a low completion percentage resulted in maintaining or decreasing the frequency or duration of sessions (with a minimum of 2 walking and 2 exercise sessions per week).

The cardiac rehabilitation nurse reviewed participant data, advised participants on weekly goals, and screened for any issues related to abnormal heart rate. Heart rate zones were modified by the nurse if new medications were prescribed or based upon a treating clinician’s advice. Each week, the cardiac rehabilitation nurse contacted the participants at a time deemed preferable by the participant to perform a cardiac rehabilitation counseling session (Multimedia Appendix 1) to review exercise and walking goal completion, check heart rate values, address concerns, and deliver standard cardiac rehabilitation modules on heart disease management (ie, smoking cessation, weight management, stress management). At the end of 12-week program, the study participant returned the health watch and tablet at a study visit and completed an exit interview, which consisted of the baseline assessment components in addition to questions regarding exercise motivation and system usability in the context of the MI-PACE program. The analytical sample for this study comprised participants who completed the full 12-week program. The Philips Internal Committee for Biomedical Experiments and the University of Massachusetts Medical School institutional review board (H00013769) reviewed and approved this study.

### Primary Study Outcomes

The primary study outcomes included measures of adherence to components of the cardiac rehabilitation system and its general usability. We examined adherence to the individual component with the following measures: health watch daily wear time in hours, proportion of completed weekly telephone counseling sessions, and proportion of exercise and walking sessions reaching the target duration. Completion of an exercise session was determined based on the number of minutes spent in or above the target exercise heart rate zone. Any exercise time spent below this heart rate zone was not considered cardiac rehabilitation exercise and was not counted toward the exercise goal. Completion of a tele-cardiac rehabilitation session was recorded by the cardiac rehabilitation nurse.

To assess the usability of the MI-PACE system, participants completed the 10-item System Usability Scale (SUS) at the end of the program [31]. Participants rated factors such as complexity, ease of use, and confidence in operating the system on a 5-point Likert-type scale (strongly disagree, disagree, neutral, agree, strongly agree). SUS scores have a range of 0 to 100, with higher scores indicating greater usability. SUS scores of 68 or higher are considered to indicate good usability [32].

### Secondary Study Outcomes

We measured participants’ daily step counts during the study period. We also determined adherence to wearing the health watch over the course of the study, by operationally defining a day of wear as registering more than 1000 steps and wearing the health watch for more than 2 hours. Participants also completed walking goals distinct from their exercise goals, defined by minutes spent walking in bouts of at least 2 minutes (regardless of them being in the target exercise heart rate zone).

Participants completed the Seattle Angina Questionnaire (SAQ) [33] to assess disease-specific quality of life at baseline and at the end of the study. The SAQ has 5 scales assessing physical limitation, angina stability, angina frequency, treatment satisfaction, and quality of life that are each scored from 0 to 100, with higher scores indicating greater disease-specific quality of life. Expert panels consider changes ≥16.0 points to be clinically meaningful [34].

### Clinical Variables

Trained study staff abstracted data on participants’ demographic, clinical, treatment, and laboratory characteristics during their index hospitalization from electronic health records—key clinical, electrocardiographic, and laboratory variables (troponin, ECG ST-segment changes, systolic blood pressure, and creatinine levels upon admission, history of renal dysfunction, Killip classification, use of diuretics, and instances of cardiac arrest)—to calculate the Global Registry of Acute Coronary Events (GRACE) risk scores a validated instrument [35], to ascertain severity of acute myocardial infarction and short-term prognosis. Study staff also abstracted baseline ECG information, exercise performance, and presence of symptoms from the stress test performed at study entry.

### Statistical Analyses

Baseline demographic and clinical characteristics for participants are presented using percentages for categorical variables, means with standard deviations for continuous variables with normal distributions, and medians with first and third quartile values for continuous variables with skewed distributions. To examine changes in cardiac rehabilitation behaviors over the course of the study, we plotted the weekly median value and interquartile ranges for participants’ mean daily health watch wear time, median and interquartile ranges of daily step count, and mean proportion of weekly exercise goals completed.

We compared baseline to end-of-study scores for the SAQ scales using Wilcoxon matched-pair signed-ranks tests due to skewed distributions.

## Results

### Sample Size and Baseline Characteristics

Overall, study staff screened 420 inpatient admissions for study eligibility, of whom 62 (15%) met eligibility criteria. Of the 62 patients who were eligible, 57 were approached, and 31 (54.4%) consented to participate and completed baseline interviews. Of these 31 individuals, participation in the cardiac telerehabilitation program was deemed to be safe for 20 individuals who successfully completed the baseline stress test (Figure 1). Two participants withdrew prematurely (one due to an unplanned vascular surgery and another because their cardiologist recommended transition to center-based cardiac rehabilitation); the remaining 18 participants (90%) completed the 12-week tele-CR program.

Of participants who completed the 12-week tele-CR program (n=18; age: mean 58 years, SD 7) (Table 1), 6 (33%) were women, 16 (89%) were non-Hispanic White individuals, 6 (33%) had been hospitalized for ST-segment elevation acute myocardial infarction, and the median GRACE score was 92.2 (IQR 82.2, 104.4). Notably, 9 (50%) and 7 (39%) participants reported travel and inadequate time, respectively, as obstacles to participating in center-based cardiac rehabilitation.

**Table 1:** Baseline characteristics of patients who completed the 12-week telerehabilitation program.

Characteristic			Value (n=18)
Age (years), mean (SD)			58 (7)
**Sex, n (%)**			
	Female	6 (33)	
	Male	12 (67)	
**Race and ethnicity, n (%)**			
	Non-Hispanic White	16 (89)	
	Middle Eastern	1 (6)	
	Unspecified	1 (6)	
BMI^a^ (kg/m^2^), median (IQR)			29.1 (27.3, 33.8)
**Clinical characteristics^b^, n (%)**			
	Atrial fibrillation or flutter	2 (11)	
	Chronic kidney disease	4 (22)	
	Depression	3 (17)	
	Diabetes	2 (11)	
	Dyslipidemia	14 (78)	
	Hypertension	9 (50)	
	Prior myocardial infarction	0 (0)	
	Obstructive sleep apnea	2 (11)	
	Currently smokes	1 (6)	
**Index hospitalization data**			
	ST-segment elevation myocardial infarction, n (%)	6 (33)	
	GRACE^c^ risk score, median (IQR)	92.2 (82.2, 104.4)	
	Maximum troponin level (mg/dL), median (IQR)	3.5 (0.8, 18.7)	
	Percutaneous coronary intervention, n (%)	14 (78)	
	Left ventricle ejection fraction (%), mean (SD)	58.8 (9.7)	
**Baseline cardiac rehabilitation stress test data**			
	Total exercise time (minutes), mean (SD)	8.4 (3.0)	
	Metabolic equivalent of task (METs), median (IQR)	10.1 (7.6, 13.4)	

^a^BMI: body mass index.

^b^Percentages do not add to 100 because patients may have more than 1 condition.

^c^GRACE: Global Registry of Acute Coronary Events.

### Adherence to Wearing Health Watch

**Figure 2:**
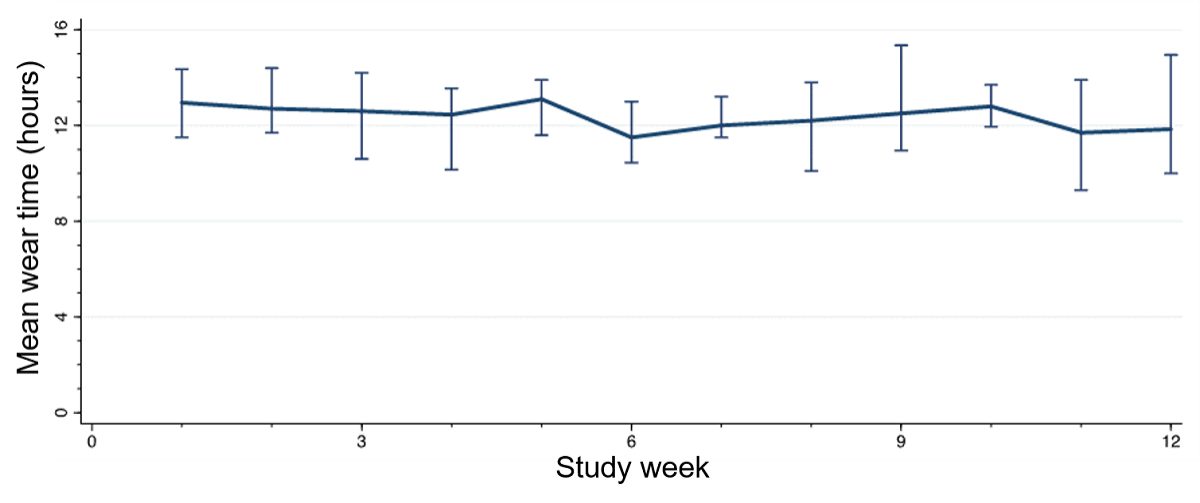
Participants' mean daily wear time by study week.

Participants wore the study health watch for a median of 61 (73%) of 84 study days (IQR 35, 78) over the 12-week study period and for a median of 12.7 hours (IQR 11.1, 13.8) per day (Table 2). Daily health watch wear time was consistent over the 12-week study period, with a mean decrease in wear time of 0.06 hours (95% CI −0.15 to 0.02 hours) per week (Figure 2).

**Table 2:** Adherence metrics to the telerehabilitation program.

Adherence metric	Value
Daily wear time, median (IQR)	12.7 (11.1, 13.8)
Days worn, median (IQR)	61 (35, 78)
Mean percentage of exercise goals met	86.0
Mean percentage of walking goals met	33.6
Mean percentage of phone sessions completed	91.7

### Exercise and Walking

**Figure 3:**
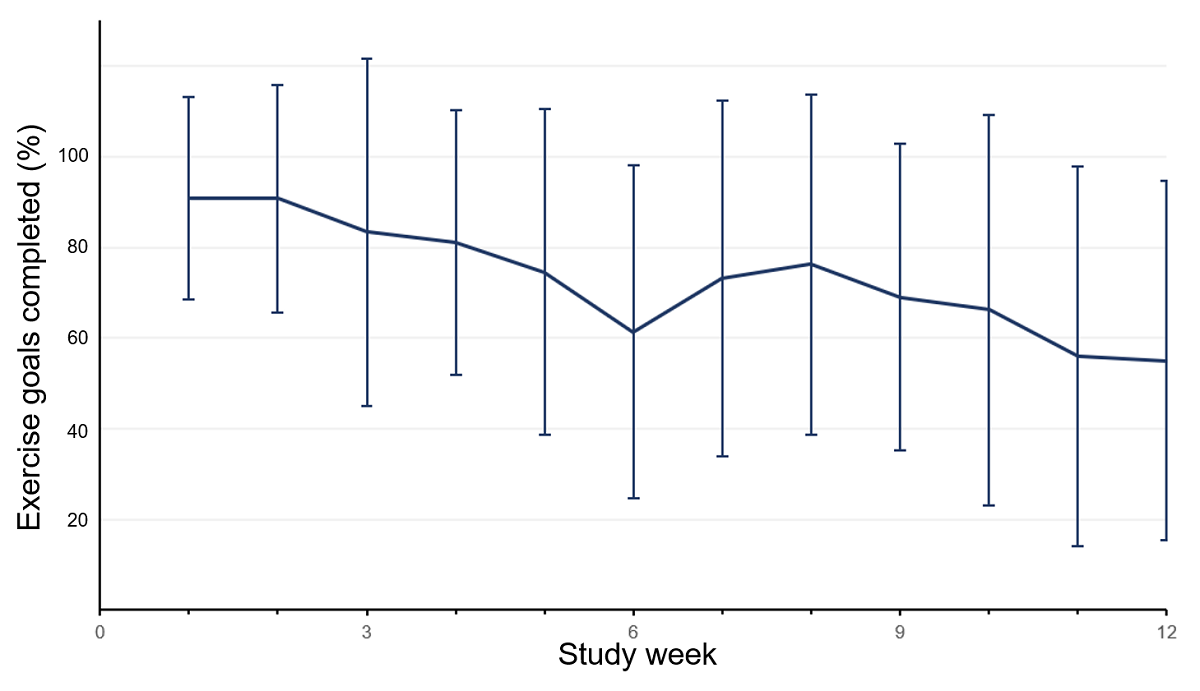
Exercise goals completed by study week.

**Figure 4:**
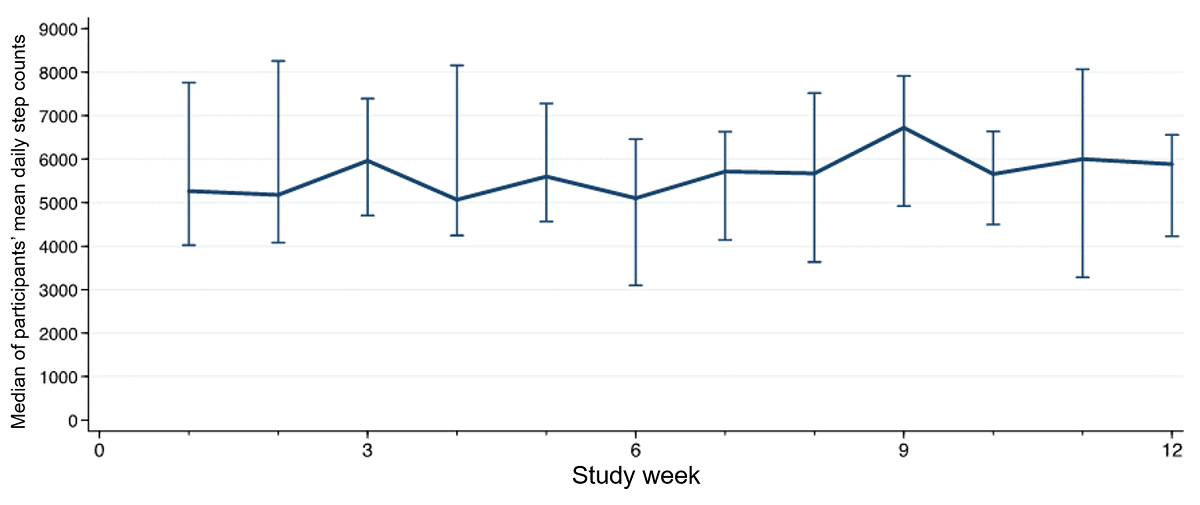
Median daily step count by study week.

Overall, participants completed a mean of 86% of prescribed exercise goals over the 12-week study period (Table 2). Participants completed a smaller proportion of exercise assignments over time, and there was an average decline of 8% (95% CI 3% to 13%) completion of prescribed exercise goals per additional study week (Figure 3). The overall percentage of completed walking goals for the entire study was 34% (Table 2). Completion of prescribed walking goals remained unchanged over the duration of the study with a mean weekly increase in completion rate of 1% (95% CI −12% to 14%) per additional week. The median of each participant’s average daily count was 6023 steps (IQR 3940, 6920). Participants’ daily step counts remained steady over the 12-week study period, with an average increase of 15 steps (95% CI −71 to 101) per additional week (Figure 4).

On average, participants walked 121 minutes per week (SD 175); the average number of walking minutes increased by 2.7 minutes per additional study week. On average participants spent 189 minutes per week (SD 210) in their exercise heart rate zone; the average number of exercise minutes decreased by 0.55 minutes per additional study week.

### Adherence to Tele-CR sessions

The lowest number of telephone counseling sessions attended by a participant was 7 out of 12, and the median number of weekly tele-CR sessions attended was 11, with 8 out of 18 participants attending all 12 sessions.

### Health-Related Quality of Life

Quality of life (*P*=.12), self-perception of physical limitation (*P*=.79), angina frequency (*P*=.67), and treatment satisfaction (*P*=.42) scores did not change significantly from baseline to study completion; however, there was a significant improvement in participants’ perceptions of their angina stability (baseline: median 50, IQR 41.7, 91.7; after tele-CR: median 100, IQR 50.0, 100; *P*=.003) (Table 3).

**Table 3:** Seattle Angina Questionnaire Quality of Life scores for survivors of an acute myocardial infarction who completed a 12-week telerehabilitation program (n=18).

Seattle Angina Questionnaire domains^a^	Score, median (IQR)		*P* value
	Baseline	12 weeks	
Physical limitation	98.6 (77.8, 100)	100 (77.8, 100)	.79
Angina stability	50.0 (50.0, 50.0)	100 (50.0, 100)	.003
Angina frequency	90.0 (80.0, 100)	100 (75.0, 100)	.67
Treatment satisfaction	100 (81.3, 100)	100 (90.6, 100)	.42
Quality of life	50.0 (41.7, 91.7)	50.0 (50.0, 91.7)	.12

^a^Each scale has a range from 0 to 100, with higher scores indicating better quality of life.

### Usability and Motivation

The median SUS score was 82.5 (IQR 65.0, 90.0). Overall, 82% of participants (14/17) reported that the system motivated them to be physically active, and 82% (14/17) also reported that the system helped them to achieve physical activity recommendations. About a third of participants agreed that, because of the system, they walked and exercised more than they previously had (Table 4).

**Table 4:** Perception of usability and motivation among survivors of an acute myocardial infarction who completed a 12-week telerehabilitation program.

Items		Participants endorsing (n=18), n (%)		
		Disagree or strongly disagree	Don’t know or neutral	Agree or strongly agree
**System usability**				
	Would use frequently^a^	0 (0)	2 (12)	15 (88)
	Found unnecessarily complex^b^	14 (88)	0 (0)	2 (13)
	Easy to use^a^	3 (18)	0 (0)	14 (82)
	Need technical support^a^	12 (71)	2 (12)	3 (18)
	Functions well integrated^a^	4 (24)	1 (6)	12 (71)
	Too much inconsistency^a^	8 (47)	0 (0)	9 (53)
	People can learn to use quickly^a^	2 (12)	1 (6)	14 (82)
	System awkward to use^a^	14 (82)	0 (0)	3 (18)
	Self-confidence using system^a^	1 (6)	2 (12)	14 (82)
	Need to learn a lot prior to use^b^	13 (81)	1 (6)	2 (13)
**Motivation**				
	The system motivates me to walk and exercise^a^	1 (6)	2 (12)	14 (82)
	The system helps me to follow the walking and exercise recommendation^a^	1 (6)	2 (12)	14 (82)
	Thanks to the system I walk and exercise more than before^a^	5 (29)	6 (35)	6 (35)

^a^Missing responses (n=1) were not included in the denominator of percentage calculations.

^b^Missing responses (n=2) were not included in the denominator of percentage calculations.

### Major Medical and Safety Events

No participants reported injuries or major adverse medical events in connection with their cardiac rehabilitation or study activities. There were no instances of recurrent cardiac events or death among participants over the course of the study.

## Discussion

### Principal Findings

In this study, we demonstrated that patients hospitalized after acute myocardial infarction who declined to participate in center-based cardiac rehabilitation participated in alternative cardiac telerehabilitation, which comprised a wearable watch, a cardiac rehabilitation app for review of exercise and walking goals, and scheduled telephone sessions with a cardiac rehabilitation nurse, found it to be acceptable. We observed moderately high rates of adherence to exercise recommendations and telephone counseling sessions.

Most indices of disease-specific quality of life remained stable over the 12-week study period; however, perceived angina stability improved. No adverse events were observed among study participants. Participants completing the 12-week cardiac rehabilitation intervention reported the cardiac rehabilitation system to be usable and motivating for physical activity. These results suggest that a nurse-assisted cardiac telerehabilitation program with an internet-connected app and wrist-based wearable device may be an acceptable alternative for patients eligible for but who decline to participate in center-based cardiac rehabilitation.

### Participation Adherence

Participants were generally adherent to the tele-CR program. Participants’ health watch wear time and step count averages remained stable throughout the entire 12-week program, which is consistent with findings of other cardiac telerehabilitation studies [4]. 

The exercise and walking goals were collaboratively set by each patient and the cardiac rehabilitation nurse and thus were individualized. Participants completed a high portion of their exercise goals. We observed a slight decline in adherence to exercise goals over the 12-week study period. These goals are based on achieving heart rate targets, and in combination with the steady step count data, our results may indicate that participants’ exercise intensities slightly declined over time. In particular, when fitness levels improve, the same exercise routine may result in a slightly lower heart rate. Another possible reason for this decrease in exercise goal adherence over the study may be because weekly exercise goals were generally increased if the previous week’s goals were met, and consequently, participants who successfully completed prior weekly exercise recommendations may have found it increasingly difficult to achieve new exercise targets.

The overall percentage of completed walking goals was lower (34%) than that that of completed exercise goals (86%), but remained constant over the 12-week study period. We hypothesize that the health watch walking time algorithm, which required that participants walk for 2 continuous minutes (no breaks lasting longer than 10 seconds) in order for the time period to be counted, may have led to underestimation of participants’ true walking time. Consistent with this hypothesis, participants in our study reported during their exit survey that interrupted or short walks were not counted toward their walking time. Despite the limitations of the approach used in our study, other activity trackers use similar approaches. For example, Fitbit counts active minutes only after 10 minutes of continuous moderate-to-intense activity [36]. In the future, we may consider lowering the walking time threshold for older adults participating in tele-CR.

Nearly half of the participants completed all 12 tele-CR sessions with the study cardiac rehabilitation nurse, and we observed high adherence to phone sessions throughout the program, though adherence was higher in the first half of the study than that in the second (95% in the first 6 weeks and 87% in the second). Although patient education is a core component of many telerehabilitation protocols [18,26,37], many models tend to utilize technology to implement passive unidirectional communication as opposed to engaging the patient in active education such as (eg, shared decision making in setting goals and assessing their overall needs with respect to their cardiac health). In fact, previous research on tele-CR that used unidirectional patient education concluded that additional telephone support and more intensive coaching would be valuable [38,39]. Our participants nearly unequivocally mentioned support from the cardiac rehabilitation nurse as a highlight of the program and as one of its most important components. The enthusiasm and patience of the cardiac rehabilitation nurse as mentioned by many of the patients is likely to be a major contributor to the high adherence rates to the cardiac rehabilitation nurse sessions.

### Usability and Motivation

Participants generally expressed enthusiasm for their experience with the MI-PACE system (SUS score: median 82.5, IQR 65.0, 90.0) and responses on motivation assessments. Recent studies [23,40,41] with tele-CR systems similar to ours reported mean SUS scores of 76 [41], 75 [40] and 65.5 [23], suggesting that tele-CR systems generally show acceptable usability despite the comorbidity and impairment burden of the target population. While the SUS has been validated for use as a complete instrument, examining its individual domains may provide specific insight into different aspects of usability. The item asking about inconsistencies in the system appears to be the only item to generate a wide distribution of responses (Table 4). This heterogeneity in response is likely due to challenges that some participants faced with walking time detection using the health watch; the fact that short and intermittent walks did not contribute to their total walking time discouraged some participants.

Participants overwhelmingly endorsed that the tele-CR system increased their motivation to perform physical activity and helped them with reaching their physical activity targets (82% for both). A much lower proportion of patients reported that the system directly increased their level of physical activity (35%). In this study, participants achieved a high level of physical activity. Participants walked for an average of 121 minutes per week (SD 175) and spending 189 minutes per week (SD 210) in their personal exercise heart rate zone. Participants’ average walking time (121 minutes per week) falls below the recommended 150 minutes of weekly moderate-intensity exercise [1], but walking time may have been underestimated by the health watch. Furthermore, the observed average of 189 minutes spent in the exercise heart rate zone is considerably greater than the 75 minutes of vigorous-intensity exercise per week recommended by the American Heart Association, which indicates that participants generally achieved adequately high levels of exercise [42].

### Disease-Specific Quality of Life

Angina stability improved significantly (*P*=.003) in tele-CR participants over the 12-week study period. The lack of statistical significance in other domains (quality of life: *P*=.12; self-perception of physical limitation: *P*=.79; angina frequency: *P*=.67; treatment satisfaction: *P*=.42) is not surprising in light of the fact that the tele-CR program was not designed to address all facets of clinical care (ie, medical treatment). Significant change in these domains is likely to require longer follow-up. It should also be noted that our study was not sufficiently powered to detect small differences in SAQ (n=18). However, our results are consistent with those of other studies [26,43] demonstrating that the effects of cardiac telerehabilitation on disease-specific and health related quality of life are mixed. For example, though it has been suggested that angina frequency may improve after 6 weeks of cardiac rehabilitation [44], other cohort studies [44,45] show no significant associations between cardiac rehabilitation participation and domains of health related quality of life. Further research is needed to elucidate component factors of cardiac rehabilitation that may contribute to changes in health related quality of life domains to clarify why they are observed in only a subset of studies.

### Telerehabilitation and Mobile Health Technology

The use of mobile and wearable technologies for heart rate and activity monitoring has revolutionized many aspects of health care, and cardiac rehabilitation is a promising area for leveraging remote monitoring to improve cardiovascular outcomes. Whereas earlier cardiac telerehabilitation programs focused on web-based portals and providing additional communications channels for patients during the rehabilitation process [17], more recent studies [18,20,21,46] have begun to utilize biosensors in wearable devices to both monitor progress and inform exercise interventions. There appears to be increasing interest in support of leveraging near-continuous biomonitoring from mobile devices to generate real-time feedback in order to individualize recommendations and maximize potential patient benefits in tele-CR. In addition, systematic reviews and meta-analyses have shown the noninferiority of home-based cardiac rehabilitation compared to traditional center-based approaches with respect to virtually all relevant outcomes [17,37], and further studies suggest that costs of tele-CR are at least comparable, if not lower than, those incurred by center-based cardiac rehabilitation [15,18,20].

Very little research, however, has focused on assessing the usability and feasibility of telemonitoring programs in a patient population fraught with a heavy burden of comorbidities (such as a cardiac rehabilitation patient population) or the organizational and systemic challenges in implementing such programs. Involving all stakeholders is important, as they are critical to successfully implement a tele-CR program. Potential differences between the number and quality of health care provider visits delivered through tele-CR versus those delivered through conventional cardiac rehabilitation further emphasize the importance of addressing human factors in tele-CR design processes [47]. Ideally, users or prescribers of the tele-CR system should be involved throughout the process, using a participatory design model, and feedback should be elicited from the patients, their caretakers, health care professionals, and health care company specialists [48]. Recently, a tele-CR intervention whose developers heavily engaged target users in the development process [49] subsequently demonstrated high ease of use (reported by participants) [23], which illustrates the need for stakeholder engagement. Involvement of all stakeholders maximizes the likelihood of designing an end product that is highly usable and addresses the needs of each relevant party to ensure successful clinical application. There is significant value in understanding the nuances surrounding the specific use of devices as well as the myriad of relevant process variables in implementation in tele-CR. This is not only an important step toward the goal of widespread dissemination and adoption of tele-CR, but arguably, a necessary one.

This study contributes to a very sparse knowledge base regarding the usability of, and adherence to, a health watch-based tele-CR program. The intervention and supportive care environment enabled by weekly interactions with a trained cardiac rehabilitation nurse and our findings from the data provided by the wearable device indicate that this specific model of tele-CR may be an acceptable alternative to center-based cardiac rehabilitation as patients overwhelmingly gave high usability ratings for the system.

### Implementation Strategy

Design of the study’s protocol necessitated careful consideration of implementation strategies in order to ensure its success. The device support provided by study staff throughout the study appears to be an important component of a positive patient experience. While participants were contacted by the study cardiac rehabilitation nurse weekly, the nurse’s role did not encompass technical support, and patients generally reported that the availability of study staff for questions regarding the watch and tablet system, both at the initial study visit as well as the duration of their enrollment, had been important to their success in use. This demonstrates the utility of having the necessary support staff to meet patient needs, both in research as well as in clinical practice, with respect to answering technical questions when they arise. One challenge that arose during implementation of this study was the need to streamline patient recruitment while respecting the clinical decisions made by their primary medical teams. Study participation required medical procedures (a stress test) not directly recommended by their medical teams, and thus, that were potentially difficult to integrate into the patient care workflow (and with consequent medical expenditures). Minimizing disruptions to routine patient care and integrating clinical research into existing health care systems is key for the success of similar projects and to ensure patient safety.

### Study Strengths and Limitations

The strengths of this study include the use of a population of patients who were eligible for cardiac rehabilitation but who declined center-based rehabilitation, the use of a tele-CR program informed by data from a validated wrist-based wearable device [29], the integration of a remote cardiac rehabilitation nurse to review data and provide standardized and scalable cardiac rehabilitation–specific health education using conventional methods of cardiac rehabilitation counseling, use of validated indices of usability and disease-specific quality of life, and objective monitoring of participants’ levels of physical activity after acute myocardial infarction. We acknowledge several limitations: the small sample size, low proportion of eligible to screened patients, and study design that focused on feasibility, adherence, and usability all contribute potential biases that may limit interpretability of the results. Selection bias may have resulted in a population with high baseline physical activity, with participants maintaining, rather than increasing, their physical fitness, which is limits generalizability to other cardiac rehabilitation populations. The study was not powered to identify individual factors associated with cardiac rehabilitation adherence or to determine the effect of cardiac rehabilitation on clinical or patient-reported outcomes (n=18). Additionally, the study cohort was relatively fit at baseline (median MET 10.6), and there may be bias with respect to the potential for activity increase over the course of the 12 weeks. Finally, we did not systematically assess contextual factors related to study implementation or physical fitness postintervention.

### Conclusions

Cardiac telerehabilitation with a wearable device for patients after acute myocardial infarction had high usability ratings as well as high rates of adherence to health watch use, exercise recommendations, and telephone counseling sessions, with no associated adverse outcomes reported. Telerehabilitation with wearables may be an alternative for patients who are eligible but unable to participate in center-based cardiac rehabilitation. Additional studies with larger patient samples are warranted to compare patient outcomes and health care resource utilization for center-based rehabilitation to those of telerehabilitation.

## Appendix

Multimedia Appendix 1 (A) Health Watch, (B) screenshot of patient-facing PACE app, (C) screenshot of patient list in the provider-facing PACE app, and (D) screenshot of heart rate graph in the provider-facing PACE app.

## Abbreviations

ECG: electrocardiogram
GRACE: Global Registry of Acute Coronary Events
SAQ: Seattle Angina Questionnaire
SUS: System Usability Scale
tele-CR: cardiac telerehabilitation
