# Supplementary material for: MI-PACE Home-Based Cardiac Telerehabilitation Program for Heart Attack Survivors: Usability Study
Source: JMIR Hum Factors. 2021 Jul 8;8(3):e18130. doi: 10.2196/18130 (PMC8299347; doi:10.2196/18130)
Supplement: Multimedia Appendix 1 [file humanfactors_v8i3e18130_app1.docx]

**A.**


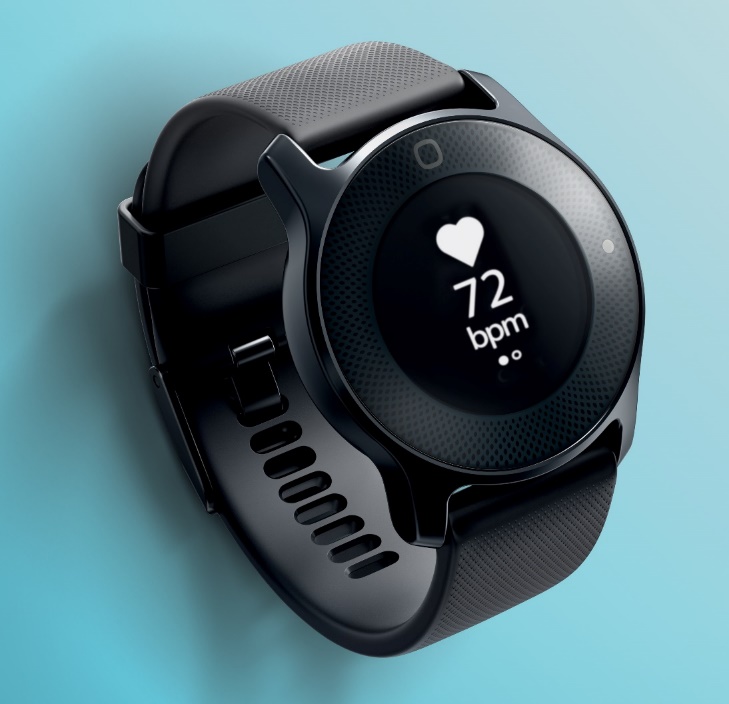


**B.**

**
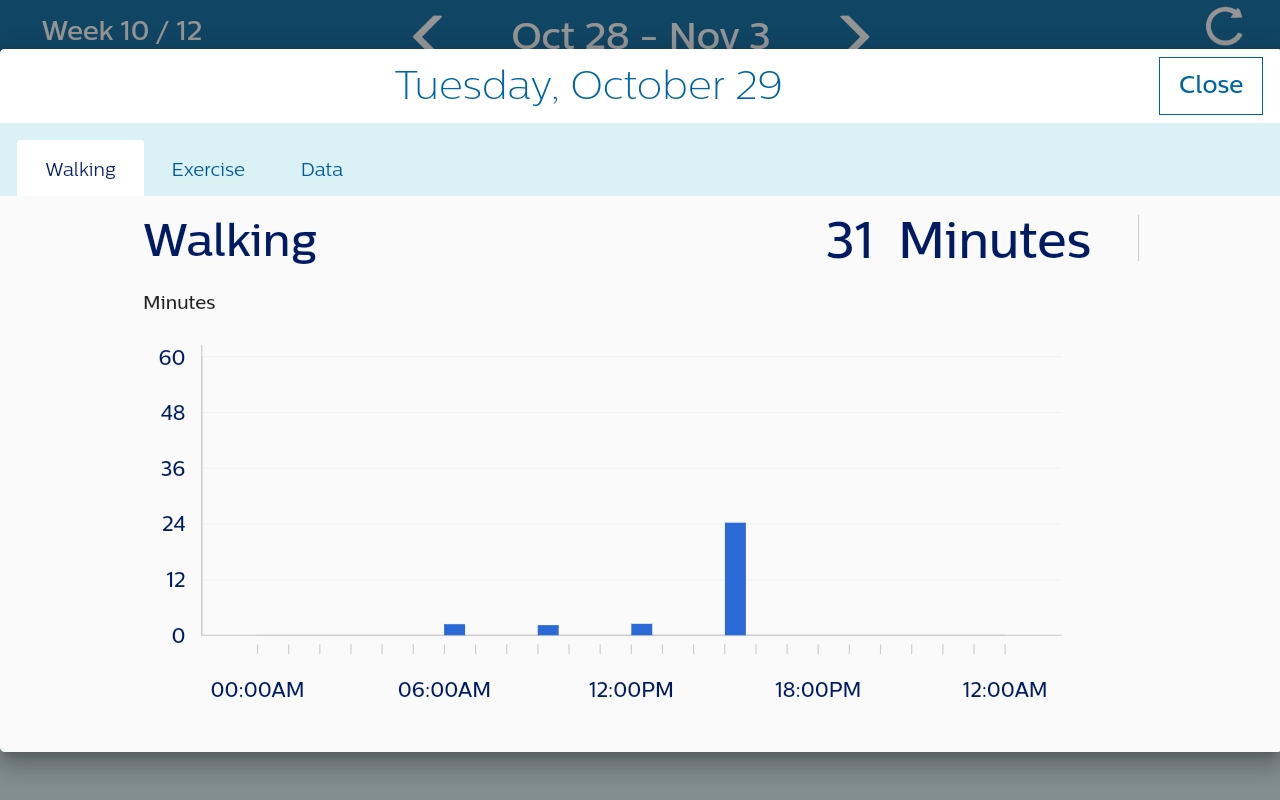
**

**C.**


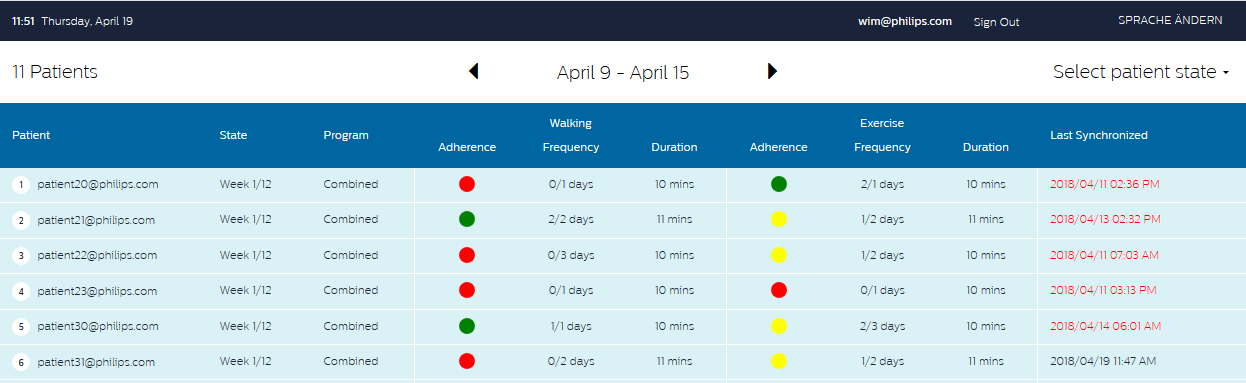


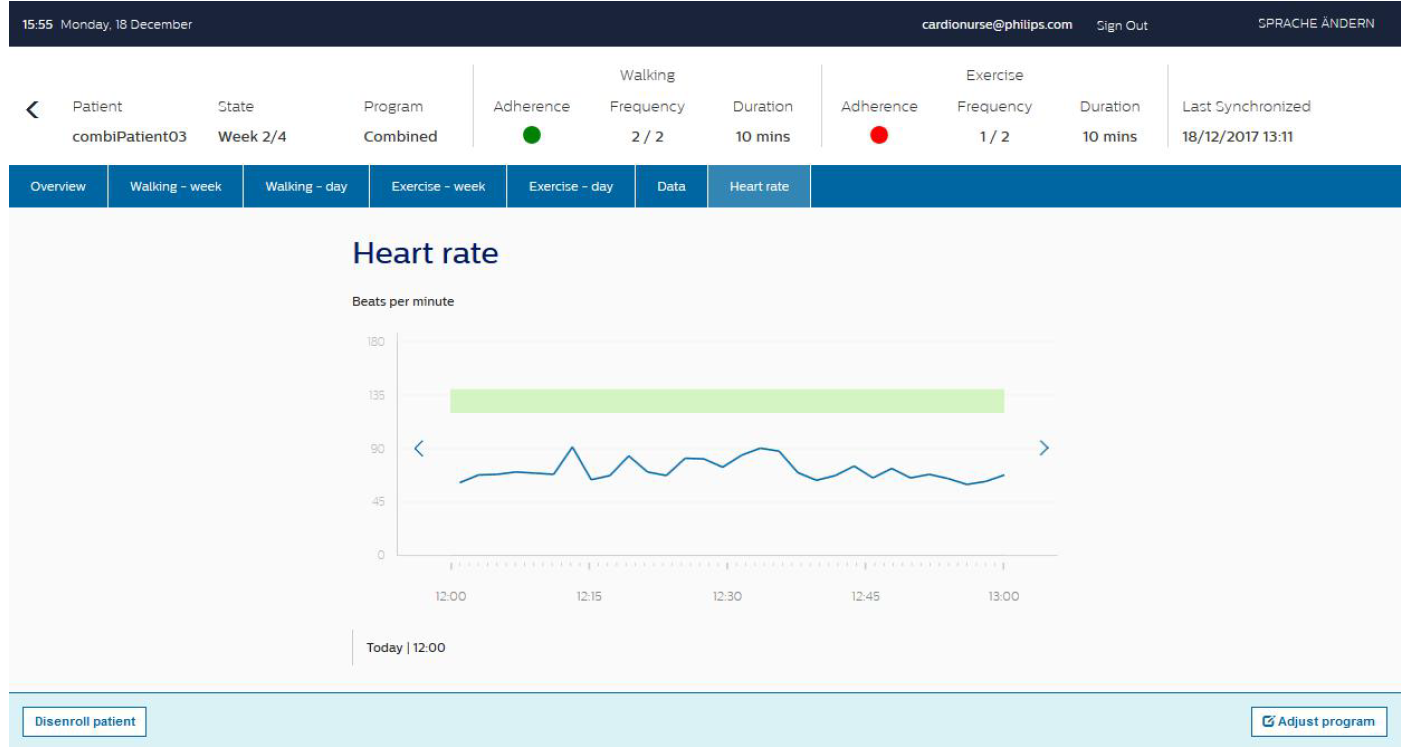
**D.**

**Figure 1: A.** Depiction of Philips® health watch **B.** Screenshot of patient-facing PACE application. **C-D.** Screenshots of patient list and heart rate graph of the provider-facing PACE application
